# Supplementary material for: Toxic and Trace Elements in Raw and Cooked Bluefish (Pomatomus saltatrix) from the Black Sea: Benefit–Risk Analysis
Source: Foods. 2026 Jan 2;15(1):140. doi: 10.3390/foods15010140 (PMC12786204; doi:10.3390/foods15010140)
Supplement: Supplementary file 1 [file foods-15-00140-s001.zip › foods-4001325-supplementary.pdf]

Table S1: Element, view mode[a], wavelength, limit of detection (LOD; µg/L), limit of quantitation (LOQ, µg/kg) and recovery values of toxic and essential elements from DORM-2 (n=7; mg/kg) using ICP-OES (n=7)

Element	Λ (nm)	LOD	LOQ	DORM -2		Recoveries (%)
				Certified values	Found values	
Cd	228.802	5.00	16.00	0.306 ±0.015	0.299±0.014	97.7
Cr	267.716	6.00	17.00	1.87±0.16	1.89 ±0.13	101.1
Cu	327.393	1.00	3.00	15.9±0.9	17.061± 0.012	107.3
Fe	238.204	1.70	5.00	341±27	345 ±18	101.2
Mn	257.610	2.00	6.10	3.66±0.34	3.71±0.21	101.4
Ni	231.604	3.00	9.00	1.36±0.22	1.29 ±0.15	94.9
Pb	220.353	5.00	16.00	0.416±0.053	0.392± 0.02	94.2
Zn	206.200	3.00	8.00	52.2±3.2	47.76 ± 1.9	91.5

[a] View mode: All samples were determined under the axial mode.

Table S2. Mineral profile of raw and cooked bluefish (in mg/kg w.w; mean±SD)

	**Raw**	**Grilled**			**Pan Fried**			**Smoked**		
			Percentage difference	Trend		Percentage difference	Trend		Percentage difference	Trend
Toxic elements										
Cd	0.024±0.001^{a}	0.029±0.012^{a}	120.8 %	↑	0.016±0.014^{a}	66.7 %	↓	0.039±0.011^{a}	162.5 %	↑
Ni	0.06±0.01^{a}	0.08±0.04^{a}	123.0 %	↑	n.d	N/A	↓	0.08±0.01^{a}	126.2 %	↑
Pb	0.14±0.05^{a}	0.34±0.20^{a}	242.9 %	↑	0.25±0.19^{a}	178.6 %	↑	0.33±0.07^{a}	235.7 %	↑
Essential elements										
Cr	0.09±0.01^{b}	0.29±0.11^{ab}	322.2 %	↑	0.10±0.09^{b}	111.1 %	↑	0.59±0.33^{a}	655.6 %	↑
Cu	0.33±0.09^{a}	0.44±0.01^{a}	133.2 %	↑	0.39±0.13^{a}	118.7 %	↑	0.38±0.08^{a}	114.5 %	↑
Fe	15.53±2.52^{a}	13.38±1.12^{a}	86.2 %	↓	12.68±0.71^{a}	81.7 %	↓	15.02±3.60^{a}	96.7 %	↓
Mn	0.82±0.01^{bc}	0.92± 0.02^{ab}	112.2 %	↑	0.55±0.21^{c}	67.1 %	↓	1.11±0.03^{a}	135.4 %	↑
Zn	14.53±0.17^{c}	16.36±0.26^{b}	112.6 %	↑	14.76±0.23^{c}	101.6 %	↑	18.75±0.32^{a}	129.0 %	↑

Legend: Mean values ± standard deviation; values in the same row that do not share a common superscript are significantly different between cooking methods ($p < 0.05$); nd—not detected; N/A—not available.

Toxic and essential elements in the cooked samples were compared with the control using relative retention values (RRV) to evaluate the effects of each cooking method. (100 %: the element is fully retained; < 100%: loss is due to cooking; > 100%: loss is possibly due to water loss or concentration effect during cooking).

Table S3. Risk values of each metal in raw and cooked bluefish samples

	THQ									TR		
	Toxic elements				*Essential elements*							
	**Cd**	**Ni**	**Pb**	**Cr**	**Cu**	**Fe**	**Mn**	**Zn**	**HI**	**Cd**	**Ni**	**Pb**
Raw	0.006	0.0001	0.001	0.001	0.001	0.402	0.0001	0.011	0.421	$5.03.10^{-7}$	$5.17.10^{-6}$	$4.19.10^{-10}$
Grilled	0.007	0.0004	0.003	0.002	0.003	0.346	0.0002	0.013	0.374	$6.08.10^{-7}$	$1.51.10^{-5}$	$1.62.10^{-9}$
Pan-fried	0.003	0.0000	0.001	0.001	0.002	0.328	0.0001	0.011	0.348	$3.14.10^{-7}$	n.d	$7.49.10^{-9}$
Smoked	0.009	0.0004	0.008	0.005	0.002	0.389	0.0002	0.015	0.428	$8.17.10^{-7}$	$1.59.10^{-5}$	$4.28.10^{-9}$

THQ= Target Hazard Quotient; HI=Hazard Index; TR=Target Risk; nd—not detected;
Calculation parameters are given in section 2.5
